# The Diet–Microbiota–Polyamine Axis in Intestinal Aging: Microbial Pathways, Functional Foods, and Physiological Implications

**DOI:** 10.3390/nu18040578

**Published:** 2026-02-10

**Authors:** Alice N. Mafe, Dietrich Büsselberg

**Affiliations:** 1 Department of Biological Sciences, Faculty of Sciences, Taraba State University, Main Campus, Jalingo 660101, Taraba State, Nigeria; mafealice1991@gmail.com; 2 Department of Physiology and Biophysics, Faculty of Medicine, Qatar Campus, Weill Cornell Medicine-Qatar, Education City, Qatar Foundation, Doha Metropolitan Area, Doha P.O. Box 22104, Qatar

**Keywords:** gut metabolites, intestinal regeneration, lactic acid bacteria, polyamine biosynthesis, age-associated dysbiosis, microbial therapeutics, anti-inflammatory metabolites, functional nutrition

## Abstract

Intestinal aging is characterized by a gradual decline in epithelial renewal capacity, barrier function, immune balance, and metabolic regulation, often accompanied by shifts in gut microbial composition. Polyamines, including putrescine, spermidine, and spermine, are vital microbial–host metabolites that support intestinal cell growth, autophagy, immune modulation, and mucosal repair. With advancing age, both host-derived and microbiota-mediated polyamine production declines, contributing to intestinal dysfunction and heightened vulnerability to inflammation and age-related disorders. This review explores the diet–microbiota–polyamine axis as a key biological framework influencing intestinal aging. It aims to integrate evidence on how dietary components and functional foods shape gut microbial ecology and, in turn, regulate microbial polyamine biosynthetic pathways that impact intestinal health. The review highlights major microbial contributors to polyamine metabolism, particularly lactic acid bacteria, and outlines mechanistic pathways linking polyamines to epithelial regeneration, inflammatory control, and gut barrier maintenance. It further discusses how age-associated dysbiosis disrupts these interactions and evaluates nutritional and microbial-based strategies such as fermented foods, prebiotics, and probiotics that may enhance polyamine availability and restore gut homeostasis. From the standpoint of food microbiology and human physiology, this synthesis underscores the translational potential of targeting microbial polyamine production through diet-based interventions. This article presents a narrative review synthesizing experimental, animal, and emerging human evidence on microbial and dietary polyamines in intestinal aging. In conclusion, modulating the diet–microbiota–polyamine axis represents a promising strategy to promote healthy intestinal aging, meriting deeper mechanistic exploration and validation through clinical studies.

## 1. Introduction

Polyamines are low-molecular-weight, positively charged compounds that are universally distributed in living organisms, with putrescine, spermidine, and spermine representing the principal forms in human biology [1,2]. These molecules are indispensable for cellular homeostasis, as they regulate nucleic acid stability, protein translation, cell growth, autophagy, and adaptive stress responses [3,4]. Within the gastrointestinal tract, polyamines are essential due to the high turnover rate of intestinal epithelial cells, where they support mucosal integrity, epithelial renewal, and tissue repair [5,6]. Intestinal polyamine pools arise from a combination of host biosynthesis, dietary sources, and microbial metabolism [7]. In the context of this review, “intestinal health” refers to the maintenance of epithelial renewal capacity, intact barrier function, balanced mucin secretion by goblet cells, regulated enteroendocrine signaling, and controlled mucosal immune responses. These processes are particularly vulnerable during aging and are central targets of polyamine-mediated regulation. A critical consideration throughout this review is that the mechanistic and physiological roles attributed to polyamines are supported by varying levels of evidence, predominantly derived from in vitro and animal studies, with human observational data and clinical intervention studies remaining comparatively limited and explicitly distinguished where applicable.

A gradual yet significant functional decline accompanies intestinal aging. Hallmark features include weakened epithelial barrier function, increased intestinal permeability, chronic low-grade inflammation, and dysregulated immune responses [8,9]. These physiological alterations are frequently paralleled by age-related gut dysbiosis, marked by reduced microbial diversity and a decline in beneficial taxa alongside an expansion of inflammation-associated microbes [10,11]. Collectively, these changes undermine gut homeostasis and contribute to systemic inflammation and the progression of age-associated diseases.

The gut microbiota is a key determinant of polyamine availability, functioning as both a producer and modulator of these metabolites [12,13]. Numerous intestinal microbes, including lactic acid bacteria and other commensals, harbor enzymatic pathways that convert dietary amino acids into bioactive polyamines [14,15]. Once produced, microbially derived polyamines can influence host signaling pathways involved in epithelial proliferation, immune regulation, and inflammatory control, establishing a direct mechanistic link between microbial metabolism and intestinal aging [16].

From the perspectives of food microbiology and human physiology, the microbiota–polyamine axis is particularly relevant because it is highly responsive to dietary modulation [17,18]. Functional foods, fermented products, probiotics, and prebiotic substrates can shape microbial community structure and enhance polyamine biosynthesis, offering accessible and low-risk strategies to maintain intestinal function across the lifespan [19,20].

This review aims to provide a comprehensive synthesis of current evidence on the diet–microbiota–polyamine axis in intestinal aging. It focuses on microbial polyamine biosynthetic pathways, diet-driven modulation of gut microbiota, and the physiological implications for intestinal health, while outlining future directions for food-based and microbial therapeutic interventions.

## 2. Polyamines: Biosynthesis, Regulation, and Functions

### 2.1. Biochemical Properties of Polyamines

Polyamines are low-molecular-weight, linear aliphatic compounds containing multiple amino groups, which render them polycationic under physiological conditions [21,22]. The principal polyamines in mammalian systems include putrescine, spermidine, and spermine, which vary in chain length and charge density, properties that determine their biological activity and molecular interactions [23,24]. Owing to their positive charge, polyamines readily bind to negatively charged macromolecules, including DNA, RNA, membrane phospholipids, and acidic proteins [25,26]. These interactions allow polyamines to influence chromatin architecture, stabilize nucleic acid structures, regulate ribosomal activity, and modulate membrane dynamics, positioning them as key regulators of cellular structure and signaling [27]. Owing to their polycationic nature, polyamines interact reversibly with negatively charged macromolecules such as DNA, RNA, and proteins through electrostatic forces, enabling rapid and flexible modulation of molecular organization in response to cellular metabolic conditions [28].

### 2.2. Microbial Versus Host Biosynthesis Pathways

**Figure 1:**
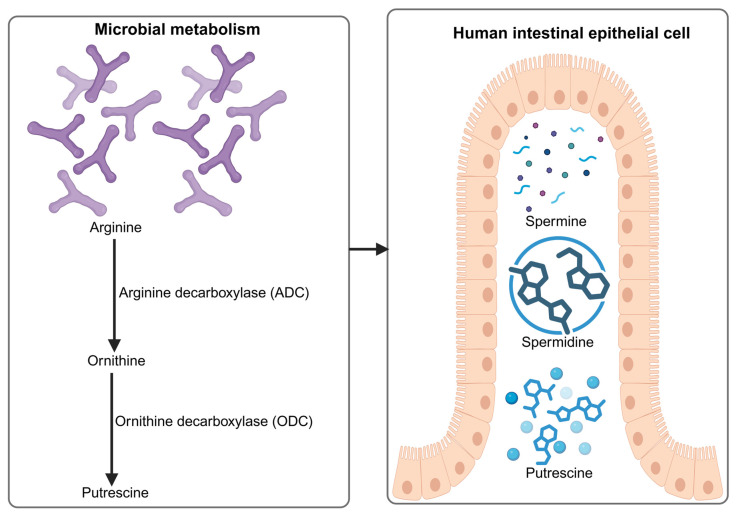
Pathways of Microbial and Host Polyamine Biosynthesis (created with BioRender, https://app.biorender.com/illustrations/695269d08651c8e950a2fb6b accessed on 9 January 2026).

Intestinal polyamine levels are controlled by a tightly regulated balance between endogenous synthesis, dietary contribution, and microbial metabolism [29]. In host tissues, polyamine biosynthesis is primarily driven by the ornithine decarboxylase (ODC) pathway, in which ornithine is decarboxylated to form putrescine, followed by sequential conversion to spermidine and spermine through aminopropyl transfer reactions [30]. Arginine also serves as an important upstream substrate, feeding into polyamine synthesis through arginine decarboxylase-dependent routes that intersect with nitrogen and amino acid metabolism [31,32]. Beyond canonical polyamines, host-derived agmatine is produced via arginine decarboxylation by arginine decarboxylase, a distinct mammalian enzyme expressed in the brain and peripheral tissues, establishing agmatine as a biologically active polyamine precursor [33]. The gut microbiota represents an additional and highly influential source of polyamines. Many intestinal bacteria harbor decarboxylase systems that convert arginine or ornithine to putrescine, with some species further synthesizing spermidine or spermine [34,35]. Microbial amino acid decarboxylation pathways include lysine decarboxylase-mediated conversion of lysine to cadaverine, which contributes to the luminal pool of biogenic amines that act as metabolite-derived signaling molecules influencing intestinal health, as demonstrated in in vitro and animal models [36]. Notably, lactic acid bacteria, *Bacteroides* sp., *Enterococcus* sp., *Escherichia* sp., *Clostridium* sp., and related taxa have been identified as contributors to luminal polyamine pools [37,38]. Figure 1 illustrates the key pathways involved in microbial- and host-derived polyamine biosynthesis, highlighting the metabolic interactions that regulate intracellular and luminal polyamine pools. Key microbial contributors to intestinal polyamine biosynthesis are outlined in Table 1. Host polyamine biosynthesis is tightly controlled by antizyme-dependent regulation of ornithine decarboxylase, with antizyme inhibitor 1 fine-tuning intracellular polyamine levels in a tissue- and stress-responsive manner [39].

### 2.3. Physiological Polyamines Versus Pathological Biogenic Amines

Physiological polyamines such as putrescine, spermidine, spermine, and agmatine are endogenously regulated metabolites essential for cellular growth and intestinal homeostasis, whereas biogenic amines including cadaverine, histamine, and tyramine mainly arise from bacterial amino acid decarboxylation in foods or dysbiotic environments, where excessive accumulation reflects impaired metabolic control and poses potential health risks [51]. The formation of biogenic amines in foods and beverages is largely driven by proteins and free amino acids, which serve as substrates for microbial or natural enzymes with decarboxylation or amination activity. Fermentation in products such as wine, beer, cider, and certain teas, as well as naturally occurring amines in some fruit-based drinks, significantly contributes to their levels, like underscoring the need for preventive strategies such as selecting low-decarboxylating starter cultures and good manufacturing practices [52]. Fermentation in alcoholic beverages, coffee, tea, and some fruit-based drinks can elevate biogenic amine levels, reflecting the activity of diverse microbial communities whose composition and metabolism influence both the safety and nutritional impact of these products [53]. Dietary exposure to biogenic amines should account for all potential sources, including foods and beverages, particularly for individuals with reduced metabolic capacity or those taking medications that may inhibit enzyme activity or interact with these compounds, increasing the risk of adverse effects [54,55]. The most effective strategy to limit biogenic amine accumulation is preventive, including the use of lactic acid bacteria strains with low decarboxylase activity and good manufacturing practices that control fermentation conditions, leveraging LAB’s natural antimicrobial and biopreservative properties [56]. From a biochemical and translational perspective, physiological polyamines and biogenic amines differ not only in their precursor amino acids, decarboxylase specificity, and regulatory control, but also in their metabolic context, bioavailability, intracellular handling, and functional targeting, reflecting distinct physiological roles and implications for disease [57]. Amino acid-derived polyamines are tightly regulated through feedback inhibition and enzyme specificity to maintain cellular homeostasis, whereas dysregulated or excessive amine production due to loss of metabolic control, can lead to local toxicity or off-target effects, as observed in microbial systems where feedback and enzyme abundance interact to ensure robust yet balanced biosynthesis [58]. This principle mirrors strategies in drug development, where amino acids are used as moieties in prodrugs to enhance bioavailability, decrease systemic toxicity, and achieve tissue-specific delivery, highlighting the importance of controlled precursor utilization. Moreover, the dynamic role of amino acids in rapid biological processes, such as neurotransmission, underscores that subtle differences in substrate availability, enzymatic control, and temporal release can have profound functional consequences [59,60,61]. Collectively, these considerations emphasize that the distinction between physiological polyamines and potentially harmful biogenic amines extends beyond molecular charge to include regulation, metabolic integration, and functional specificity.

## 3. Intestinal Aging: Biology, Pathophysiology, and Microbiota Changes

### 3.1. Features of the Aging Intestine

**Figure 2:**
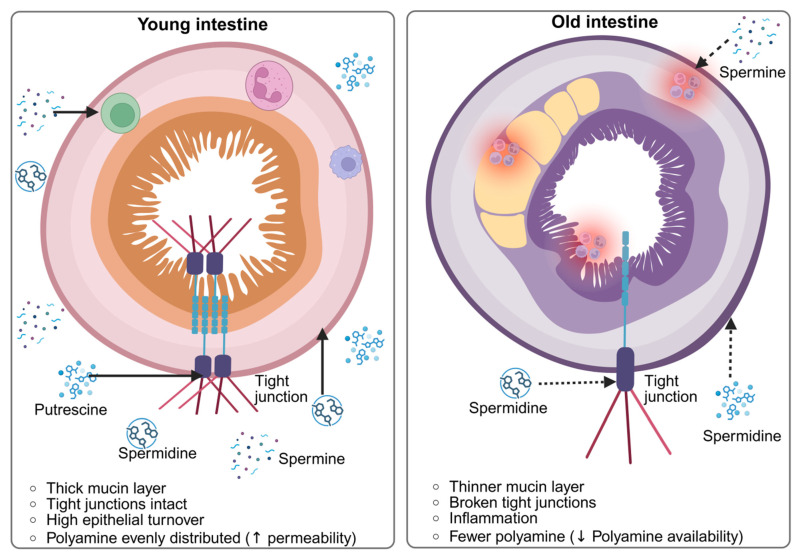
“Young” vs. “Aged” Intestine: Sites of Polyamine Action (created with BioRender). https://app.biorender.com/illustrations/695275e5717757622b97a4f9 accessed on 9 January 2026.

Aging is associated with notable structural and functional changes in the gastrointestinal tract. A key characteristic of the aging intestine is a decline in epithelial renewal, mainly due to reduced intestinal stem cell activity and slower crypt cell proliferation. This limits the epithelium’s capacity to replace damaged cells and maintain tissue homeostasis [62,63]. Concurrently, aging is associated with enhanced intestinal permeability, often described as a “leaky gut,” resulting from altered expression and organization of tight junction proteins. Increased permeability facilitates the passage of microbial components and dietary antigens into the underlying tissue [64,65]. In addition, the mucus barrier becomes thinner and less effective with age, reducing its protective role against microbial contact with the epithelium. These cumulative changes contribute to the development of persistent low-grade inflammation, or inflammaging, characterized by sustained immune activation and elevated pro-inflammatory mediators that further impair intestinal function and resilience [66,67]. Figure 2 compares the “young” and “aged” intestinal environments, emphasizing age-dependent differences in polyamine availability, absorption sites, and biological activity along the gut epithelium.

### 3.2. Age-Related Changes in the Gut Microbiota

The gut microbiota undergoes marked compositional and functional shifts during aging. One of the most consistent patterns is the beneficial microbial taxa, including species that produce protective metabolites. This loss is often accompanied by an increased prevalence of pathobionts and opportunistic bacteria that can promote inflammatory responses and epithelial stress. Together, these reduce microbial diversity and diminish metabolic flexibility in the gut ecosystem [68,69]. Beyond compositional changes, aging also affects microbial metabolic capacity. Age-associated dysbiosis is associated with reduced production of microbially derived polyamines, reflecting both the loss of key producer species and disrupted microbial cross-feeding networks. As a result, the metabolic dialogue between the microbiota and host becomes less efficient, negatively influencing intestinal health [70].

### 3.3. Declining Polyamine Availability During Aging

Aging is accompanied by a gradual reduction in polyamine levels within the intestine and systemically. This decline is partly due to decreased endogenous synthesis, stemming from reduced activity of polyamine biosynthetic enzymes and age-related metabolic constraints. In parallel, intestinal uptake of polyamines may be impaired as epithelial transport mechanisms and absorptive capacity deteriorate with age [71,72]. Dietary habits also play a critical role in shaping polyamine availability in older populations. Reduced intake of polyamine-rich foods and fermented products, together with changes in appetite and food diversity, can further limit exogenous polyamine supply. When combined with microbial dysbiosis and impaired host synthesis, these factors create a state of relative polyamine deficiency, which may accelerate intestinal aging and exacerbate inflammation and barrier dysfunction [73,74].

## 4. Microbial Polyamines in the Aging Gut

### 4.1. Probiotic and Commensal Sources

The gut microbiota constitutes a major reservoir of bioavailable polyamines, a role that becomes increasingly important with aging as endogenous polyamine synthesis declines. Several probiotic and commensal microorganisms contribute directly or indirectly to intestinal polyamine pools [75,76]. Lactic acid bacteria (LAB) [77], widely distributed in fermented foods and commonly employed as probiotics [78,79,80], are among the most prominent producers. These organisms can synthesize putrescine and spermidine either through intrinsic metabolic pathways or via cooperative interactions with other gut microbes [81]. *Bifidobacteria*, which are often less abundant in older adults, also play a key role in maintaining polyamine homeostasis. Through amino acid metabolism and cross-feeding interactions, these bacteria support the production of spermidine and spermine [82,83]. Additionally, genera such as *Enterococcus* sp., *Bacillus* sp., and *Streptococcus* sp. possess polyamine biosynthetic capabilities, highlighting the collective contribution of commensal microbial networks to sustaining intestinal polyamine availability during aging [84].

### 4.2. Mechanistic Actions of Microbial-Derived Polyamines in Intestinal Aging

Microbial-derived polyamines promote intestinal homeostasis and resilience during aging by coordinating epithelial renewal, barrier integrity, immune balance, and stress-response pathways, with preclinical evidence indicating their ability to counteract age-related microbial and inflammatory imbalances [85]. A key role of microbial polyamines is to enhance epithelial proliferation and regeneration in the aging intestine. By promoting cell cycle progression, migration, and differentiation, polyamines support mucosal repair and sustain epithelial turnover processes that decline with age. Preclinical in vitro and in vivo studies show that polyamine supplementation can restore regenerative capacity in aged epithelia [86]. Microbial polyamines support intestinal barrier integrity by regulating the expression, localization, and assembly of tight and adherens junction proteins, including E-cadherin, and by modulating cytoskeletal organization. These actions reinforce paracellular sealing, limit age-related increases in intestinal permeability, and are largely demonstrated in preclinical and in vitro models [87,88].

Beyond structural support, microbial-derived polyamines help maintain intestinal immune homeostasis by modulating innate and adaptive immune cell activity, regulating cytokine production, and influencing redox-sensitive signaling pathways. These metabolites restrain excessive inflammation and preserve mucosal balance, with evidence primarily from in vitro and preclinical studies [89,90]. Autophagy is a key pathway through which microbial polyamines, particularly spermidine, modulate intestinal aging. By stimulating autophagic flux, spermidine enhances the clearance of damaged proteins and organelles, supports epithelial stem cell function, reinforces barrier integrity, and increases cellular resilience to inflammatory and metabolic stress. These effects are well-documented in vitro and in animal models, with emerging observational support in humans [91]. Collectively, these mechanistic actions position microbial polyamine metabolism as a central regulator of intestinal physiology during aging. Experimental evidence strongly supports roles in epithelial repair, barrier maintenance, immune modulation, and autophagy, yet direct confirmation in humans remains limited and largely observational. Further clinical and interventional studies are needed to validate the translational potential of microbial-derived polyamines for promoting healthy intestinal aging.

### 4.3. Dysbiosis and Pathogenic Overproduction of Harmful Polyamines

Although commensal microbes generate beneficial polyamines, dysbiosis may promote the overproduction of potentially harmful biogenic amines, including putrescine and cadaverine, which are often produced by spoilage-associated or pathogenic bacteria. These compounds typically arise from uncontrolled amino acid decarboxylation and may accumulate in the gut or in inadequately processed or stored foods [92,93]. Cadaverine, generated from lysine by microbial lysine decarboxylase (CadA/Ldc), is enriched in dysbiotic communities where it depletes lysine essential for epithelial renewal, promotes butyrate-producing microbiota, and drives barrier dysfunction and inflammatory signaling, as shown in in vitro and animal models [94,95]. In older individuals, diminished detoxification capacity and compromised intestinal barrier function can heighten susceptibility to the adverse effects of these amines, such as mucosal irritation, inflammatory responses, and systemic toxicity. These observations underscore the importance of maintaining microbial balance and implementing strict control over food fermentation and storage practices in aging populations [96].

## 5. Dietary Polyamines and Food Sources Relevant to Aging

### 5.1. Polyamine-Rich Foods

Dietary intake represents a major source of polyamines, with numerous foods naturally containing putrescine, spermidine, and spermine [97,98]. Fermented products such as yogurt, cheese, fermented vegetables, and traditional fermented foods are particularly rich due to microbial activity during fermentation [99,100]. Fruits, vegetables, mushrooms, and seafood also contribute appreciable amounts, with polyamine content influenced by species, maturity, and processing methods [101]. Food processing and storage conditions substantially affect polyamine concentrations. Factors such as heat treatment, fermentation duration, and hygienic handling can either enhance beneficial polyamine levels or favor the formation of undesirable biogenic amines, emphasizing the need for controlled processing strategies [102,103]. Dietary sources of polyamines and their relative abundance are summarized in Table 2.

### 5.2. Microbial Fermentation as a Natural Enrichment Strategy

Microbial fermentation provides a sustainable method for enriching foods with beneficial polyamines. Starter culture selection is crucial, as microbial strains differ markedly in their capacity to synthesize or degrade these compounds. Appropriately selected lactic acid bacteria and fermentative microbes can increase spermidine content while minimizing the accumulation of harmful amines [116,117]. From a food microbiology perspective, fermentation parameters including substrate composition, temperature, pH, and duration, strongly shape polyamine profiles. Optimization of these factors enables the production of fermented foods with enhanced safety and functional value, particularly for aging consumers [118].

### 5.3. Polyamines as Functional Food Components for Healthy Aging

Accumulating evidence indicates that polyamines, particularly spermidine, function as bioactive dietary components relevant to healthy aging. Higher intake of spermidine has been associated with increased lifespan and improved metabolic and cardiovascular outcomes in both experimental and observational studies. Dietary polyamines, particularly spermidine, act as functional food components that support healthy aging by promoting autophagy, enhancing intestinal and cardiovascular function, and modulating immune and metabolic homeostasis. Evidence arises from preclinical models and human observational studies [119]. Within the aging gut, polyamines modulate immune responses, promote autophagy, and support epithelial integrity [120]. These intestinal effects have been demonstrated primarily through mechanistic and preclinical studies (in vitro and animal/in vivo), highlighting how microbial polyamines support gut barrier integrity, modulate inflammation, and maintain metabolic and immune homeostasis which play major processes that decline with aging and influence susceptibility to age-related diseases [121,122]. Moreover, dietary polyamines may attenuate chronic inflammation and oxidative stress, the major drivers of intestinal and systemic aging. Evidence for the inflammation- and oxidative stress-modulating effects of polyamines is largely based on mechanistic and preclinical studies, showing regulation of reactive oxygen species, antioxidant defenses, and inflammatory signaling, with direct clinical validation still limited [123,124]. Incorporating polyamine-rich foods and carefully designed fermented products into daily diets, therefore, represents a promising functional nutrition strategy to support gut health, immune equilibrium, and overall physiological resilience in older adults [125,126]. However, human evidence remains largely observational, highlighting the need for controlled dietary intervention trials to confirm translational benefits (clinical/interventional).

### 5.4. Dietary Amino Acid Precursors and Substrate Availability for Microbial Polyamine Synthesis

Dietary and endogenous proteins that escape small intestinal digestion provide the principal nitrogenous substrates for microbial polyamine biosynthesis in the large intestine. Between 3 and 11 g of such proteins and peptides reaches the colon daily, where bacterial proteases and peptidases release amino acids for microbial use. Among these, arginine, ornithine, and lysine are particularly important: arginine can be converted by gut bacteria into agmatine and subsequently putrescine, ornithine serves directly as a substrate for putrescine through decarboxylation, and lysine metabolism generates cadaverine. These amino acid-dependent pathways highlight how the recycling of host-derived proteins links dietary intake, microbial metabolism, and colonic polyamine availability, supporting both microbial growth and intestinal physiological functions [127]. Aging is accompanied by physiological changes that affect protein digestion and intestinal transit, including reduced pancreatic enzyme output, altered mastication due to dentition loss, delayed gastric emptying, and slower gut motility. Consequently, a larger fraction of dietary protein reaches the colon in older adults, increasing substrate availability for microbial proteolytic fermentation. This promotes enhanced amino acid catabolism by the gut microbiota and may elevate local production of polyamines and related metabolites. Evidence from both human and animal studies indicates decreased postprandial amino acid absorption and reduced fecal protein digestion in elderly populations, reflecting combined effects of host digestive decline and age-associated shifts in microbial community composition [128].

While increased microbial polyamine synthesis can support epithelial maintenance and stress resilience under eubiotic conditions, excessive proteolytic fermentation, especially in fermented foods, can lead to the accumulation of potentially harmful biogenic amines and inflammatory by-products. Dietary strategies, including high-quality protein sources, balanced amino acid intake, and the use of plant-derived additives in fermented products, can help limit biogenic amine formation by inhibiting microbial decarboxylases and controlling microbial activity. This duality underscores the importance of combining dietary protein management with microbial regulation to optimize polyamine profiles and maintain gut health during aging [129]. Importantly, these observations suggest that microbial-derived polyamines represent a dynamic interface between diet and host physiology, complementing endogenous host synthesis and direct dietary polyamine intake in determining overall polyamine exposure during aging.

## 6. Biophysical and Biophysiological Actions of Polyamines in the Aging Intestine

Polyamines influence intestinal aging through fundamental biophysical and biophysiological mechanisms operating at the molecular, cellular, and tissue levels. As aging is accompanied by reduced cellular repair capacity and heightened vulnerability to stress, the structural and regulatory properties of polyamines become increasingly critical for maintaining intestinal integrity [130]. Their cationic nature enables direct interactions with negatively charged biomolecules, allowing polyamines to function as stabilizers, metabolic modulators, and signaling intermediates within aging intestinal tissues [131]. Such molecular interactions have been characterized mainly in biochemical and cellular systems (in vitro). A key molecular function of polyamines is their ability to stabilize DNA and RNA, thereby supporting cellular repair and renewal. This nucleic acid-stabilizing role is supported primarily by mechanistic and preclinical evidence (in vitro and animal/in vivo). By binding nucleic acids, polyamines reduce susceptibility to oxidative damage and structural destabilization, hallmarks of aging cells. This interaction facilitates proper chromatin organization and preserves transcriptional and translational fidelity, ultimately sustaining epithelial turnover and limiting genomic instability in the aging intestine [132].

It is important to recognize that the biological effects of polyamines in the aging intestine result from the combined contributions of host synthesis, dietary intake, and microbial production, each with context-dependent roles. Host-derived polyamines maintain systemic and intracellular pools essential for basal cellular functions, including proliferation, differentiation, and tissue maintenance, while dietary polyamines absorbed primarily in the upper gastrointestinal tract, support circulating and tissue-associated levels. Emerging evidence also suggests that these integrated polyamine sources may influence age-related conditions, such as bone loss, sarcopenia, and epithelial decline, highlighting their broader significance in healthy aging [133]. In contrast, microbially derived polyamines are produced locally in the colon, where they act primarily on colonic epithelial cells and mucosal immune components to maintain barrier integrity and epithelial–microbiota homeostasis. This localized contribution becomes increasingly important during aging, when reduced digestive efficiency, shifts in microbial composition, and declining epithelial resilience heighten reliance on microbiota-mediated polyamine production for intestinal stability [134].

Polyamines, particularly spermidine, also play a pivotal role in restoring autophagic activity, which declines with age. Spermidine-induced autophagy has been consistently demonstrated in vitro and in animal models, where it preserves cellular and tissue homeostasis, and its relevance is indirectly supported by human observational studies linking autophagy dysfunction to a wide range of age-related disorders, including cardiovascular, neurodegenerative, metabolic, and musculoskeletal diseases [135]. By modulating epigenetic regulators and nutrient-sensing pathways, spermidine enhances the clearance of damaged proteins and organelles. In intestinal epithelial cells, this autophagic activation promotes barrier renewal, supports stem cell function, and improves resistance to inflammatory and metabolic stress, thereby contributing to tissue homeostasis during aging [136,137]. These intestinal outcomes have not yet been conclusively confirmed in clinical intervention studies. At the level of the epithelial barrier, polyamines reinforce membrane stability and tight junction integrity. This effect has been demonstrated primarily in well-controlled experimental intestinal models, including in vitro and animal studies, which provide mechanistic insights into gut functionality, epithelial health, and host–microbiota interactions [138]. Their interactions with membrane phospholipids enhance bilayer organization and reduce oxidative vulnerability, while their regulatory effects on cytoskeletal dynamics support tight junction assembly and maintenance. These actions are particularly relevant in older individuals, in whom increased intestinal permeability is associated with chronic inflammation and systemic metabolic disturbances [139,140].

**Figure 3:**
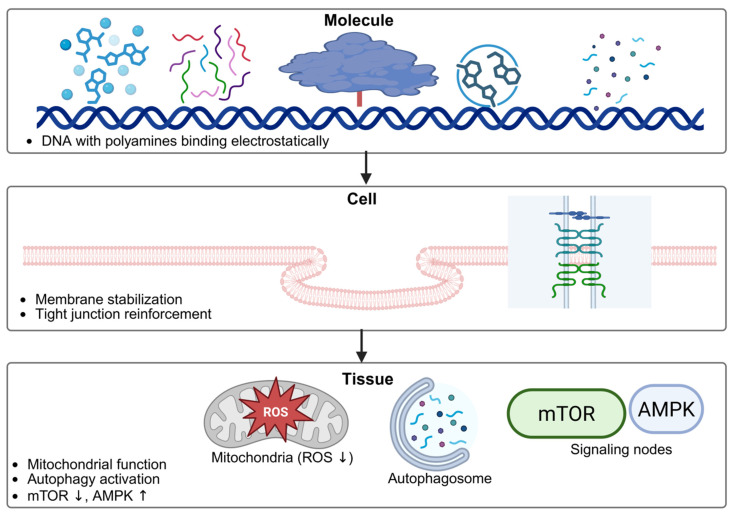
Biophysical mechanisms of polyamine action at cellular and molecular levels (created with BioRender https://app.biorender.com/illustrations/695275f96834159ac0a654dc accessed on 7 January 2026).

Polyamines further contribute to mitochondrial preservation by stabilizing mitochondrial membranes, supporting efficient oxidative phosphorylation, and limiting excessive generation of reactive oxygen species. Through these mechanisms, they help maintain cellular energy balance and protect epithelial cells from oxidative injury. Evidence for mitochondrial protection is derived primarily from in vitro and animal studies, which demonstrate how interventions can preserve mitochondrial structure, regulate bioenergetics, and prevent dysfunction associated with aging and disease, including neurodegenerative, cardiovascular, metabolic, and cancer-related conditions [141]. In parallel, polyamines influence inflammatory signaling by modulating redox-sensitive pathways and cytokine networks, thereby dampening low-grade chronic inflammation commonly observed in aging intestines [142,143]. These anti-inflammatory effects are supported mainly by preclinical data. Crucially, polyamines intersect with key longevity-associated signaling pathways, such as mTOR and AMPK. Regulation of immune and autophagy-related pathways by spermidine has been demonstrated primarily in experimental models, where it promotes CD4^+^ T-cell differentiation toward regulatory phenotypes in vitro and enhances gut Treg cell populations in mice, with protective effects that depend on intact autophagic machinery [144]. By attenuating mTOR activity and promoting AMPK signaling, spermidine supports metabolic flexibility, stress adaptation, and autophagy induction. This coordinated regulation of nutrient-sensing pathways enables intestinal cells to better adapt to age-related metabolic challenges [145]. Figure 3 depicts the biophysical mechanisms of polyamine action at cellular and molecular levels, including their interactions with nucleic acids, membranes, ion channels, and protein synthesis machinery. The mechanisms illustrated are derived predominantly from preclinical studies. The influence of polyamines on established biomarkers of intestinal aging is presented in Table 3.

## 7. Diet–Microbiota–Polyamine Interactions in Intestinal Aging

**Figure 4:**
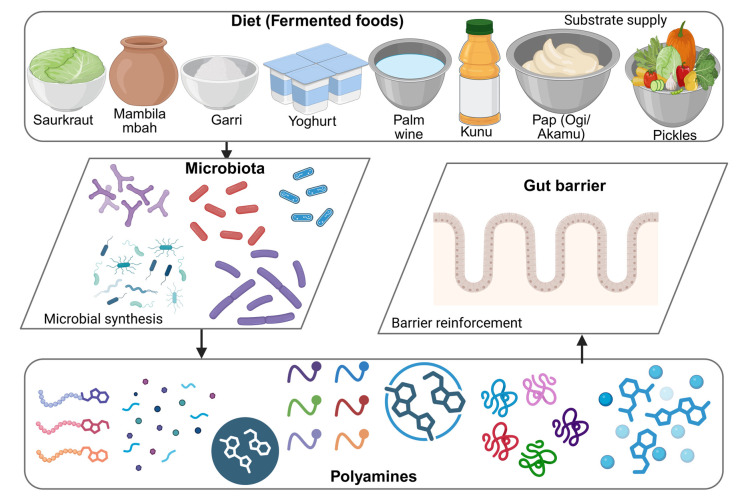
Diet–Microbiota–Polyamine Axis (created with BioRender https://app.biorender.com/illustrations/69527fcd132544d6ca0ec95e accessed on 9 January 2026).

Intestinal polyamine availability in older adults emerges from a dynamic interplay between dietary intake, microbial metabolism, and host physiology [159]. This integrative concept is supported primarily by mechanistic animal studies and human observational data, as controlled interventions remain limited and often do not account for sex-specific differences in microbiota-mediated metabolic and immunological responses during aging [160]. Dietary patterns strongly shape microbial composition and functional capacity, determining the extent to which gut microbes synthesize and transform polyamines [161]. Most evidence for diet-driven modulation of microbial polyamine production comes from animal studies and human observational cohorts (in vivo and observational). While dietary changes can rapidly shift gut microbial composition, such taxonomic alterations do not always translate to changes in metabolic function; emerging data suggest that polyamine biosynthesis and other microbial activities can vary independently of bacterial abundance, with implications for host metabolic and immune health [162]. Polyamines produced by certain bacterial strains can be secreted into the intestinal lumen and used by other microbes that lack the biosynthetic capacity to produce them, highlighting metabolic cross-feeding. Beyond basic cellular roles, microbial polyamines also contribute to biofilm formation, stress resistance, bacteriocin and toxin activity, and survival within hostile environments, emphasizing their multifaceted functional significance in the gut ecosystem [163]. During aging, disruptions in these cooperative metabolic interactions may exert greater functional consequences than compositional alterations alone, leading to impaired polyamine availability despite relatively preserved microbial diversity. Diets rich in plant-based foods, fermentable substrates, and traditional fermented products favor microbial communities capable of sustaining polyamine production [164]. These interactions operate through reciprocal feedback mechanisms in which microbial-derived polyamines enhance epithelial barrier function and immune regulation, thereby creating a favorable niche for beneficial microbiota. Such feedback mechanisms have been demonstrated primarily in experimental models (in vitro and animal/in vivo), where polyamines have been shown to mediate key processes in cell growth, differentiation, and stress responses. Insights from plant studies, particularly in *Arabidopsis thaliana*, highlight the essential role of polyamines in embryogenesis and organogenesis, emphasizing their tightly regulated spatial and temporal activity [165]. Disruption of this axis through dietary insufficiency or microbiota imbalance can weaken polyamine-mediated protection and accelerate intestinal aging. This integrated framework highlights diet as a modifiable factor for restoring microbial polyamine metabolism and supporting intestinal resilience in later life. Figure 4 summarizes the diet–microbiota–polyamine axis, demonstrating how dietary substrates, microbial metabolism, and host regulatory pathways converge to influence polyamine homeostasis and health outcomes. Human intervention studies validating this axis remain limited.

## 8. Safety Considerations, Toxicology, and Food Industry Relevance

Although physiologically essential, excessive accumulation of certain polyamines and biogenic amines poses toxicological risks, particularly for elderly individuals with diminished metabolic clearance and compromised gut barriers. Evidence for adverse effects of biogenic amines arises primarily from toxicological studies and clinical observations rather than controlled dietary trials. High concentrations of histamine, tyramine, and putrescine in foods can cause intoxication, with symptoms influenced by dose and potential synergistic interactions, underscoring the need for risk assessment and regulation [166]. Elevated exposure may provoke gastrointestinal irritation, inflammatory responses, or systemic effects, emphasizing the need to define safe intake thresholds for aging populations [167]. In food systems, polyamine levels also serve as markers of microbial activity and product quality. This application is supported by food microbiology and analytical research, including in vitro studies and industrial-scale observations, demonstrating that microbial fermentation, bioproducts, and biotransformation processes enhance food safety, nutritional functionality, and shelf life in sustainable food systems [168]. High concentrations of putrescine and cadaverine are frequently associated with spoilage and inadequate hygienic practices, especially in protein-rich and fermented foods. Consequently, polyamine profiling is increasingly recognized as a valuable tool for assessing food safety and freshness [169]. Regulatory oversight of polyamine content remains inconsistent across regions. Advances in analytical technologies, including chromatographic and sensor-based detection methods, have improved the accuracy of monitoring polyamine levels. These technologies have been validated primarily in laboratory and industrial settings rather than clinical contexts. Incorporating these tools into food safety frameworks will be essential for maximizing functional benefits while minimizing health risks [170]. It is important to distinguish between physiologically beneficial polyamines, such as spermidine, spermine, and agmatine, which support epithelial renewal, barrier integrity, and immunomodulation, and potentially harmful biogenic amines, including putrescine and cadaverine when produced in excess or during dysbiosis. Beneficial polyamines enhance intestinal resilience by supporting epithelial regeneration, proteostasis, and protein synthesis, and, as demonstrated in vivo in old mice, dietary or microbial modulation of polyamine pathways can restore age-related declines in tissue repair, highlighting their potential to promote healthy intestinal aging [171], whereas toxic biogenic amines can build up when fermentation is poorly controlled, food spoils, or certain microbes with amino acid-decarboxylating activity overgrow, potentially triggering inflammation, epithelial damage, and adverse health effects [172,173]. This dual perspective emphasizes the need for balanced microbial activity, careful selection of fermentation processes, and monitoring of polyamine content in foods to optimize both safety and functional benefits. A distinction between beneficial and toxic polyamines relevant to food systems is provided in Table 4.

## 9. Research Gaps and Emerging Opportunities

Despite substantial experimental evidence, human data on polyamine supplementation and dietary intake in aging populations remain limited. Future clinical studies should prioritize elderly cohorts and integrate microbiome, metabolomic, and functional health outcomes to establish evidence-based recommendations. Food innovation strategies aimed at optimizing polyamine profiles through controlled fermentation, processing, and storage are also needed. Additionally, the development of probiotic strains with targeted polyamine-producing capabilities presents a promising avenue for functional nutrition. At the mechanistic level, advanced biophysical approaches, including molecular modeling and simulation studies, may further elucidate polyamine interactions with membranes, nucleic acids, and signaling proteins, strengthening translational relevance.

## 10. Conclusions

Polyamines play a central role in preserving intestinal structure and function during aging through coordinated molecular, metabolic, and signaling mechanisms. As endogenous synthesis declines with age, dietary sources and microbial metabolism become critical determinants of polyamine availability. Mechanistic insights demonstrate that polyamines support nucleic acid stability, autophagy, epithelial barrier integrity, mitochondrial function, and immune balance, processes fundamental to intestinal repair and resilience. These findings underscore the potential of polyamine-focused dietary and microbial strategies for developing functional foods tailored to aging populations. By integrating perspectives from food science, microbiology, and biophysiology, polyamines emerge as key modulators of intestinal aging and as promising targets for nutritional interventions that promote healthy longevity.

## Figures and Tables

**Table 1:** Microbial Species Involved in Polyamine Biosynthesis.

Microbial Group	Representative Species	Major Polyamines Produced	Biosynthetic Pathway	Relevance to Aging Gut	Strain-Dependent Variability	Evidence Type	Reference
Lactic acid bacteria (LAB)	*Lactobacillus plantarum*, *L. casei*	Putrescine, spermidine	Arginine/ornithine decarboxylation	Widely used probiotics; safe and diet-accessible	High—production capacity varies markedly between strains	In vitro; fermented food models	[40,41]
*Bifidobacteria*	*B. longum*, *B. adolescentis*	Spermidine, spermine	Amino acid metabolism & cross-feeding	Declines with age; key target for intervention	Moderate—influenced by strain genetics and substrate availability	In vitro; animal/in vivo	[42]
*Enterococci*	*Enterococcus faecium*	Putrescine	Ornithine decarboxylase	Dual role: beneficial or opportunistic	High—some strains are associated with excessive amine production	In vitro	[43,44]
*Bacillus* sp.	*B. subtilis*	Spermidine	Polyamine synthase pathways	Relevant in fermented foods	Moderate—dependent on fermentation conditions and strain selection	In vitro; food fermentation studies	[45,46]
*Streptococci*	*Streptococcus thermophilus*	Putrescine	Arginine metabolism	Common dairy starter culture	Low–moderate—strain differences reported	In vitro; dairy fermentation models	[47,48]
Pathobionts	*Proteus* sp., *Clostridium* sp.	Putrescine, cadaverine	Unregulated decarboxylation	Linked to dysbiosis and toxicity	High—strain-specific overproduction under dysbiotic conditions	In vitro; animal/in vivo	[49,50]

Note: Polyamine production capacity varies substantially at the strain level and cannot be generalized at the species level; environmental conditions and substrate availability further modulate biosynthetic output.

**Table 2:** Polyamine Content in Common Foods.

Food Category	Representative Foods	Dominant Polyamines	Relative Content	Notes Relevant to Aging	Reference
Fermented dairy	Yogurt, cheese	Spermidine, putrescine	High	Content depends on starter cultures and ripening	[104]
Fermented vegetables	Sauerkraut, kimchi	Putrescine, spermidine	High	Can be optimized through controlled fermentation	[105,106]
Legumes & whole grains	Soybeans, lentils, wheat germ	Spermidine	Moderate–High	Major contributors in plant-based diets	[107,108]
Fruits & vegetables	Citrus fruits, tomatoes, spinach	Putrescine	Low–Moderate	Content varies with maturity	[109]
Mushrooms	Button, shiitake	Spermidine, spermine	Moderate	Emerging functional food interest	[110,111]
Seafood	Fish, shellfish	Spermine, putrescine	Variable	Sensitive to storage and spoilage	[112,113]
Processed meats	Sausages, cured meats	Putrescine, cadaverine	High (undesirable)	Associated with spoilage and safety concerns	[114,115]

Note: Polyamine content in fermented foods is highly dependent on starter culture composition, strain selection, fermentation conditions, and storage duration.

**Table 3:** Effects of Polyamines on Intestinal Aging Biomarkers.

Aging-Related Biomarker	Effect of Polyamines	Dominant Polyamine	Mechanistic Basis	References
DNA integrity	Protection and stabilization	Spermidine, spermine	Electrostatic binding to nucleic acids	[146,147]
Epithelial turnover	Increased proliferation	Putrescine	Cell cycle regulation	[148]
Autophagic flux	Upregulation	Spermidine	mTOR inhibition, epigenetic modulation	[149,150]
Tight junction integrity	Preservation	Spermidine	Cytoskeletal and protein assembly support	[151,152]
Mitochondrial function	Enhanced efficiency	Spermidine	Reduced ROS, membrane stabilization	[153,154]
Inflammatory tone	Suppression	Spermidine, spermine	NF-κB and cytokine modulation	[155,156]
Intestinal permeability	Reduced (“leaky gut”)	Spermidine	Barrier reinforcement	[157,158]

**Table 4:** Beneficial versus Toxic Polyamines in Food Systems.

Polyamine/Amine	Primary Source	Physiological Role	Risk at High Levels	Food Safety Relevance	References
Spermidine	Diet, microbiota	Longevity, autophagy	Low toxicity	Target compound for functional foods	[119,174]
Spermine	Diet, host cells	DNA stabilization	Minimal risk	Naturally regulated	[175]
Putrescine	Microbes, foods	Cell proliferation	GI irritation	Indicator of spoilage	[176,177]
Cadaverine	Spoilage bacteria	None (toxic by-product)	Histamine potentiation	Major food safety concern	[178,179]
Histamine	Spoiled fish, fermented foods	Neuroactive	Hypertension, headaches	Regulated biogenic amine	[180,181]

## Data Availability

No new data were created or analyzed in this study. Data sharing is not applicable to this article.

## Abbreviations

The following abbreviations are used in this manuscript:

AMPK	Adenosine Monophosphate-Activated Protein Kinase
DNA	Deoxyribonucleic Acid
LAB	Lactic Acid Bacteria
mTOR	Mechanistic Target of Rapamycin
NF-κB	Nuclear Factor Kappa B
ODC	Ornithine Decarboxylase
RNA	Ribonucleic Acid
ROS	Reactive Oxygen Species
